# Geospatial Socioeconomic Indicators and Penicillin Allergy Delabeling in Primary Care Patients

**DOI:** 10.1001/jamanetworkopen.2025.28714

**Published:** 2025-08-22

**Authors:** Kimberly G. Blumenthal, Andrew J. King, Valerie E. Stone, Stephen Bartels, Dylan T. Norton, Madelyn L. Eippert, Yuqing Zhang, Alysse Wurcel

**Affiliations:** 1 Division of Rheumatology, Allergy, and Immunology, Department of Medicine, Massachusetts General Hospital, Boston; 2 Harvard Medical School, Boston, Massachusetts; 3 Rheumatology and Allergy Clinical Epidemiology Research Center, The Mongan Institute, Boston, Massachusetts; 4 Brigham and Women’s Hospital, Boston, Massachusetts; 5 Tufts University School of Medicine, Boston, Massachusetts; 6 Division of Geographic Medicine and Infectious Diseases, Tufts Medical Center, Boston, Massachusetts; 7 Division of General Internal Medicine, Boston Medical Center, Boston, Massachusetts

## Abstract

This cross-sectional study evaluates the association between socioeconomic indicators and penicillin allergy delabeling prevalence in Boston, Massachusetts.

## Introduction

Penicillin allergy labels in patient electronic health record (EHR) data often do not represent true or persistent allergies.^1^ Prescribing alternative (non–β-lactam) antibiotics increases antimicrobial resistance (AMR), adverse effects, health care costs, and mortality.^1^ Removing penicillin allergy mislabels, or delabeling, is an evidence-based strategy permitting β-lactam antibiotic prescribing.^2^ Clinicians delabel patients by evaluating allergy history, removing erroneous entries, and/or procedures like drug challenges. Given that AMR infections disproportionately impact economically disadvantaged populations,^3^ we assessed socioeconomic indicators’ association with penicillin allergy delabeling prevalence.

## Methods

We used EHR data, including self-reported demographics and delabel status,^4^ to identify primary care patients with a penicillin allergy record from January 2019 to April 2022 at Mass General Brigham (MGB) and Tufts Medicine (TM). Merging patient zip code onto geospatial data, we calculated validated indicators: the social vulnerability index (SVI, total and subscales: socioeconomic status [SES], household characteristics, racial and ethnic minority status, and housing type and transportation) and American Community Survey uninsured and unemployment rates, as well as median household income (eTable in Supplement 1). This study was approved by MGB and TM institutional review boards with a waiver of informed consent because study design was retrospective and as such, research could not practically be carried out without the waiver. Reporting followed Strengthening the Reporting of Observational Studies in Epidemiology (STROBE) reporting guidelines.

We assessed delabeling prevalence across the greater Boston area, comparing highest 10 vs lowest 10 per capita income zip codes using analysis of variance with Bonferroni adjustment for multiple comparisons. We computed penicillin allergy delabeling prevalence across each indicator’s quartile (Q) then compared the lowest Q with each subsequently higher Q using prevalence ratios with 95% CIs from generalized estimating equation regression models. Poisson distribution and log-link function accounted for patient-level clustering within zip codes. There were minimal missing data (eMethods in Supplement 1). Geospatial zip code analysis used R and RStudio version 4.4.2 (R Project for Statistical Computing). Statistical analyses were conducted in SAS version 9.4 (SAS Institute), with a 2-sided *P* < .05 considered significant.

## Results

Of 76 709 patients (46 625 [61%] MGB, mean [SD] age 56 [18] years, 53 129 [69%] female, 3564 [4%] Asian, 4261 [6%] Black, 61 802 [81%] White, 7082 [9%] other [American Indian or Alaska Native or missing race], 5617 [7%] Hispanic ethnicity, 4021 [5%] non-English language preference), 5195 (6.8%) were delabeled. The mean delabeling prevalence difference between highest and lowest income areas was 6.4 percentage points (*t*_1_ = 3.2; *P* = .004) (Figure).Delabeling was less likely in those more socially vulnerable compared with less socially vulnerable areas, and in lower SES vs higher SES areas (*P* for trend all <.001) (Table). Delabeling prevalence was also lower in areas with higher uninsured and unemployment rates and lower median income (*P* for trend all <.001).

**Figure.**
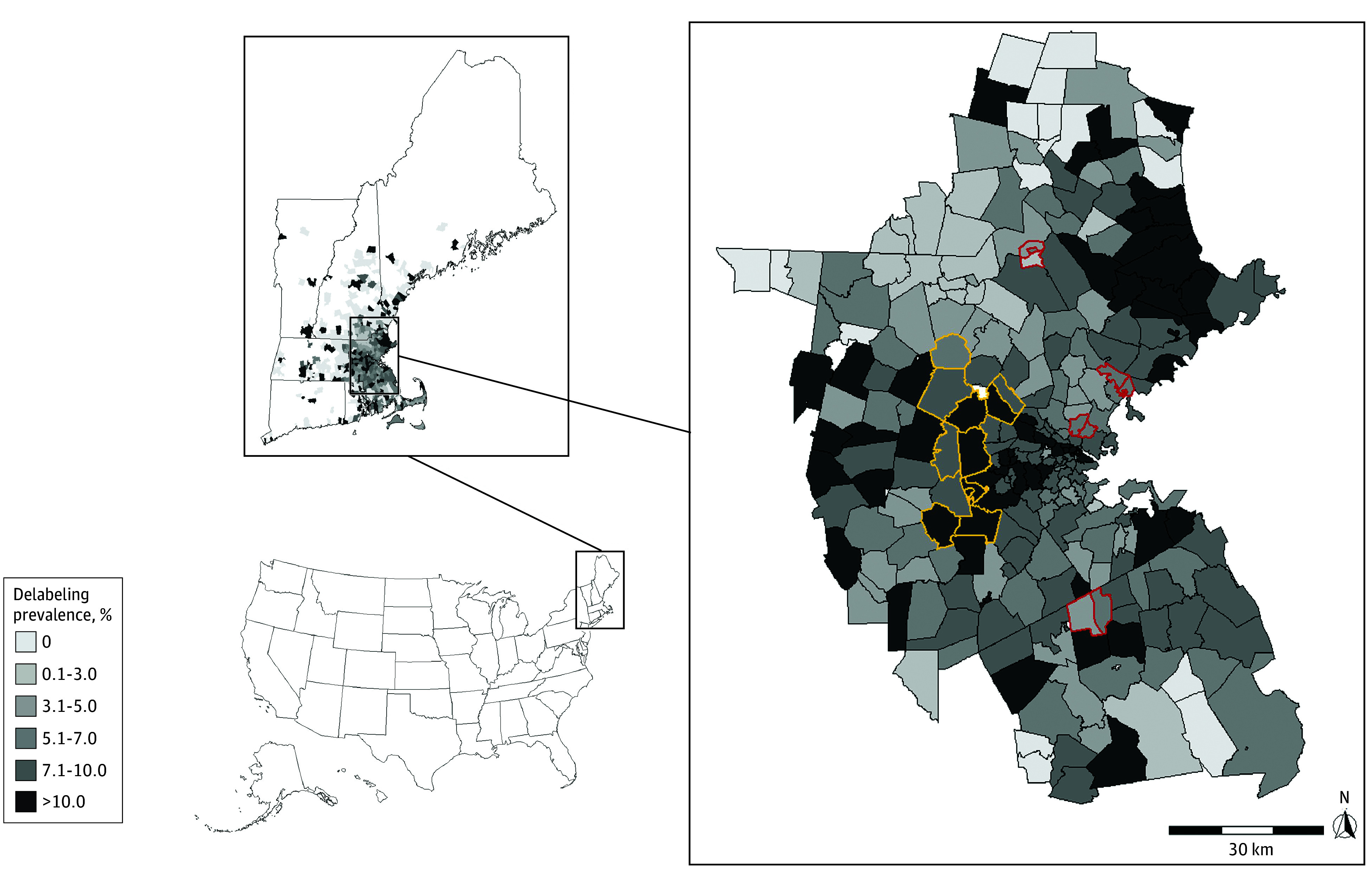
Geospatial Map of Penicillin Allergy Delabeling Prevalence Zip code areas for New England (upper left) and Greater Boston Area (right) with map of US for context (bottom left). Areas outlined in yellow represent the 10 highest income zip code areas in Massachusetts towns, including Weston (02493), Dover (02030), Wellesley (02482), Sherborn (01770), Lexington (02420 and 02421), Carlisle (01741), Lincoln (01773), Concord (01742), and Wayland (01778) with income per capita ranges from $150 253 to $354 387 in 2022 US dollars. Areas outlined in red represent the 10 lowest income zip code areas in Massachusetts towns including Lawrence (01840, 01841, and 01843), Chelsea (02150), Everett (02149), Brockton (02301 and 02302), and Lynn (01901, 01902, and 01904) with income per capita ranges from $17 984 to $23 099 in 2022 US dollars. The mean penicillin allergy delabeling prevalence was 6.4 percentage points higher, on average, in the 10 highest per capita income areas compared with the 10 lowest per capita income areas (*P* = .004). Only zip code areas with more than 3 observations are shown. Income per capita used the Massachusetts Department of Revenue for fiscal year 2022. Geospatial zip code tabulation areas are from the 2020 TIGER/Line Shapefiles: Zip Code Tabulation Areas from the US Census Bureau, Geography Division.

**Table.** Socioeconomic Geospatial Indicators and Association With Penicillin Allergy Delabeling Prevalence

Indicator and Q-based categories^a^	No. total	No. delabeled	Delabeling prevalence (95% CI)	Prevalence ratio (95% CI)		*P* value for trend^c^
				Unadjusted	Adjusted^b^	
Social Vulnerability Index (SVI), rankings						
SVI total						
Q1	18 901	1344	7.11 (6.74-7.48)	1 [Reference]	1 [Reference]	<.001
Q2	19 466	1337	6.87 (6.51-7.22)	0.97 (0.90-1.04)	1.00 (0.93-1.08)	
Q3	19 023	1471	7.73 (7.35-8.11)	1.09 (1.01-1.17)	0.94 (0.87-1.01)	
Q4	19 319	1043	5.40 (5.08-5.72)	0.76 (0.70-0.82)	0.85 (0.79-0.93)	
SVI SES						
Q1	19 135	1560	8.15 (7.76-8.54)	1 [Reference]	1 [Reference]	<.001
Q2	19 221	1351	7.03 (6.67-7.39)	0.86 (0.80-0.92)	0.93 (0.87-0.99)	
Q3	19 059	1239	6.50 (6.15-6.85)	0.80 (0.74-0.86)	0.84 (0.78-0.90)	
Q4	19 294	1045	5.42 (5.10-5.74)	0.66 (0.62-0.72)	0.80 (0.74-0.87)	
SVI household characteristics						
Q1	19 141	1343	7.02 (6.65-7.38)	1 [Reference]	1 [Reference]	.02
Q2	19 213	1405	7.31 (6.94-7.68)	1.04 (0.97-1.12)	1.01 (0.94-1.08)	
Q3	19 178	1313	6.85 (6.49-7.20)	0.98 (0.91-1.05)	0.98 (0.91-1.06)	
Q4	19 177	1134	5.91 (5.58-6.25)	0.84 (0.78-0.91)	0.91 (0.84-0.98)	
SVI racial and ethnic minority status						
Q1	19 178	1235	6.44 (6.09-6.79)	1 [Reference]	1.03 (0.96-1.12)	.55
Q2	19 176	1276	6.65 (6.30-7.01)	1.03 (0.96-1.11)	1.08 (1.00-1.16)	
Q3	19 135	1570	8.20 (7.82-8.59)	1.27 (1.19-1.37)	0.95 (0.87-1.03)	
Q4	19 220	1114	5.80 (5.47-6.13)	0.90 (0.83-0.97)	1.03 (0.96-1.12)	
SVI housing type and transportation						
Q1	19 172	1243	6.48 (6.13-6.83)	1 [Reference]	0.97 (0.90-1.05)	.60
Q2	19 183	1255	6.54 (6.19-6.89)	1.01 (0.94-1.09)	0.99 (0.92-1.07)	
Q3	18 724	1335	7.13 (6.76-7.50)	1.10 (1.02-1.18)	0.97 (0.90-1.05)	
Q4	19 630	1362	6.94 (6.58-7.29)	1.07 (0.99-1.15)	0.97 (0.90-1.05)	
American Community Survey Measures						
Uninsured rate						
Q1	18 965	1517	8.00 (7.61-8.39)	1 [Reference]	1 [Reference]	<.001
Q2	19 475	1449	7.44 (7.07-7.81)	0.93 (0.87-1.00)	0.99 (0.93-1.06)	
Q3	19 319	1186	6.14 (5.80-6.48)	0.77 (0.71-0.83)	0.89 (0.82-0.96)	
Q4	18 950	1043	5.50 (5.18-5.83)	0.69 (0.64-0.74)	0.79 (0.73-0.85)	
Unemployment rate						
Q1	19 098	1568	8.21 (7.82-8.60)	1 [Reference]	1 [Reference]	<.001
Q2	19 249	1295	6.73 (6.37-7.08)	0.82 (0.76-0.88)	0.97 (0.90-1.04)	
Q3	19 236	1186	6.17 (5.83-6.51)	0.75 (0.70-0.81)	0.85 (0.79-0.92)	
Q4	19 126	1146	5.99 (5.66-6.33)	0.73 (0.68-0.79)	0.83 (0.77-0.90)	
Median family income						
Q1	18 781	1146	6.10 (5.76-6.44)	1 [Reference]	1 [Reference]	<.001
Q2	19 569	1248	6.38 (6.04-6.72)	1.05 (0.97-1.13)	1.05 (0.97-1.14)	
Q3	19 205	1241	6.46 (6.11-6.81)	1.06 (0.98-1.14)	1.11 (1.02-1.20)	
Q4	19 154	1560	8.14 (7.76-8.53)	1.33 (1.24-1.44)	1.30 (1.20-1.40)	

Abbreviations: Q, quartile; SES, socioeconomic status; SVI, social vulnerability index.

^a^ See eTable in Supplement 1. Q4 indicates greater area deprivation for all indicators except median family income, where Q4 represents less area deprivation.

^b^ Adjusted model controlled for patient age, sex, race, ethnicity, and hospital system (Mass General Brigham vs Tufts).

^c^ *P* value for trend is derived from the significance of a single continuous variable with values set to the within quartile median in a separate adjusted model that changes the functional form of each geospatial variable from categorical to continuous.

## Discussion

We found reduced penicillin allergy delabeling in lower income areas; in adjusted analyses, reduced delabeling was associated with higher social vulnerability, uninsured and unemployment rates, and lower SES and median household income. These data suggest delabeling access or uptake is not equitable across SES, even among demographically similar patients at the same primary care institutions.

Limited observational data suggest White, non-Hispanic individuals of higher SES more likely carry penicillin allergy labels.^2^ This study demonstrates lower rates of delabeling in vulnerable populations among those eligible for delabeling while controlling for individual demographics. Given antibiotic prescribing disparities^5^ and AMR infection disparities,^3^ improving equitable access to penicillin allergy delabeling might effectively improve infectious diseases outcomes.

We used data from 2 health care systems in greater Boston, thus limiting generalizability to areas with different population characteristics and underrepresenting rural settings. Also, geospatial indicators are highly correlated, so we only adjusted for individual patient confounders. Ideal penicillin allergy label assessment would consider the temporal relationship between allergic reaction, label placement, and its subsequent removal, but EHR allergy data are cross-sectional, and label entry does not correspond to reaction date.

Across a large Boston-based multisite primary care cohort, penicillin allergy delabeling prevalence was lowest in areas of social vulnerability and economic deprivation, suggesting inequitable delabeling for those economically disadvantaged. Future research should identify barriers and facilitators to delabeling and evaluate access expansion strategies for vulnerable communities.

## Notes

Supplement 1. **eTable. **Geospatial Economic Indicators **eMethods. **Additional Information on Assessment of Missing Zip Code Data
eReferences.

Supplement 2.
Data Sharing Statement
