# Supplementary material for: Geospatial Socioeconomic Indicators and Penicillin Allergy Delabeling in Primary Care Patients
Source: JAMA Netw Open. 2025 Aug 22;8(8):e2528714. doi: 10.1001/jamanetworkopen.2025.28714 (PMC12374214; doi:10.1001/jamanetworkopen.2025.28714)
Supplement: Supplement 1. — eTable. Geospatial Economic Indicators eMethods. Additional Information on Assessment of Missing Zip Code Data eReferences. [file jamanetwopen-e2528714-s001.pdf]

Supplemental Online Content

Blumenthal KG, King AJ, Stone VE, et al. Geospatial socioeconomic indicators and penicillin allergy delabeling in primary care patients. *JAMA Netw Open*. 2025;8(8):e2528714. doi:10.1001/jamanetworkopen.2025.28714

eTable. Geospatial Economic Indicators

eMethods. Additional Information on Assessment of Missing Zip Code Data

eReferences

This supplemental material has been provided by the authors to give readers additional information about their work.

© 2025 Blumenthal KG et al. *JAMA Network Open*.

eTable. Geospatial Economic Indicators

Indicator	Definition	Citations	Data Source[a]
**Social Vulnerability Index, rankings**			
Social Vulnerability Index overall percentile ranking (SVI Total)	Social vulnerability refers to the demographic and socioeconomic factors that adversely affect communities that encounter hazards and other community-level stressors.<br><br>Higher values indicate greater area deprivation.<br><br>The source variables from the U.S. Census data used in this measure are the following: Civilian unemployment 61+ years; no high school diploma 25 + years; below 150% poverty rate; housing cost burden (households spending >=30% of annual income on housing); no health insurance; persons 65+ years; persons 17 years and younger; persons 5+ years with a disability; single-parent households with children under 18 years; persons 5+ years who speak English less than well; persons who are racial and/or ethnic minorities (total population minus non-Hispanic white); housing with 10+ units; mobile homes; households with more people than rooms; households with no vehicle access; persons who are in institutional and non-institutional group homes.	Flanagan et al. *Journal of Homeland Security and Emergency Management* (2011)[1] ; Brignone et al. *JAMA Network Open* (2024)[2]	Centers for Disease Control and Prevention and Agency for Toxic Substances and Disease Registry (CDC/ATSDR)[3]<br><br>Data extracted 2020.
Social Vulnerability Index percentile ranking for socioeconomic sub-scale (SVI SES)	Higher values indicate greater area deprivation.<br><br>Socioeconomic Status variables include measures of: Below 150% Poverty rate; Unemployed rate; Housing Cost Burden; No High School Diploma Rate; and No Health Insurance rate.		
Social Vulnerability Index percentile ranking for household characteristics sub-scale	Higher values indicate greater area deprivation.<br><br>Household Characteristics variables include measures of: Aged 65 & Older; Aged 17 & Younger; Civilian with a Disability; Single-Parent Households; English Language Proficiency.		
Social Vulnerability Index percentile ranking for Racial & Ethnic Minority Status sub-scale	Higher values indicate greater area deprivation.<br><br>Racial & Ethnic Minority Status variables include measures of: Hispanic or Latino (of any race); Black or African American, Not Hispanic or Latino; Asian, Not Hispanic or Latino; American Indian or Alaska Native, Not Hispanic or Latino; Native Hawaiian or Pacific Islander, Not Hispanic or Latino; Two or More Races, Not Hispanic or Latino; Other Races, Not Hispanic or Latino.		

© 2025 Blumenthal KG et al. *JAMA Network Open*.

Indicator	Definition	Citations	Data Source[a]
Social Vulnerability Index percentile ranking for Housing Type & Transportation sub-scale	Higher values indicate greater area deprivation.<br><br>Housing Type & Transportation variables include measures of: Multi-Unit Structures; Mobile Homes; Crowding; No Vehicle; Group Quarters.		
**American Community Survey Measures**			
Uninsured Rate	Proportion of civilian population age 19-64 with no health insurance coverage only<br><br>Higher values indicate greater area deprivation.	McIntyre et al. *JAMA Health Forum* (2024)[4]	U.S. Census Bureau American Community Survey Table for Health Insurance Coverage[5]<br><br>Data extracted 2021.
Unemployment Rate	Unemployment Rate for the Population 16 Years and Over.<br><br>Higher values indicate greater area deprivation.	Azagba et al. *BMC Psychiatry* (2021)[6]	U.S. Census Bureau American Community Survey[7]<br><br>Data extracted 2021.
Median Family Income	Median household income in the past 12 months.<br><br>Higher values indicate lower area deprivation	Udalova et al. *JAMA* (2022)	U.S. Census Bureau American Community Survey[7]<br><br>Data extracted 2021.

[a] Geospatial data were downloaded at the lowest level of geography available and aggregated to 2020 US census zip-code equivalents (i.e., Zip Code Tabulation Areas [ZCTAs]) using the Census.gov relationship conversion files. American Community Survey (ACS) variables were aggregated to ZCTAs from block group level (FIPS12 codes), SVI variables from census tract level (FIPS11 codes), and County health rankings from county level (FIPS5 codes).

[b] Mass General Brigham patient zip codes were from November 2023 and Tufts Medicine patients zip codes were from January 2024.

© 2025 Blumenthal KG et al. *JAMA Network Open*.

eMethods. Additional Information on Assessment of Missing Zip Code Data

There were n=338 patients with missing zip-codes in the electronic health record data that were excluded from this analysis; the penicillin allergy de-labeling rate was not different among the excluded vs included sample (5.6% vs 6.8%; χ^2_1=0.7, p=.40).

eReferences

1. Flanagan BE, Gregory EW, Hallisey EJ, Heitgerd JL, Lewis B. A Social Vulnerability Index for Disaster Management. *Journal of Homeland Security and Emergency Management*. 2011;8(1)
2. Brignone E, LeJeune K, Mihalko AE, Shannon AL, Sinoway LI. Self-Reported Social Determinants of Health and Area-Level Social Vulnerability. *JAMA Network Open*. 2024;7(5):e2412109-e2412109. doi:10.1001/jamanetworkopen.2024.12109
3. ATSDR. SVI Data & Documentation Download. https://www.atsdr.cdc.gov/place-health/php/svi/svi-data-documentation-download.html
4. McIntyre A, Smith RB, Sommers BD. Survey-Reported Coverage in 2019-2022 and Implications for Unwinding Medicaid Continuous Eligibility. *JAMA Health Forum*. 2024;5(4):e240430-e240430. doi:10.1001/jamahealthforum.2024.0430
5. U.S. Census Bureau. American Community Survey Tables for Health Insurance Coverage. https://www.census.gov/data/tables/time-series/demo/health-insurance/acs-hi.html
6. Azagba S, Shan L, Qeadan F, Wolfson M. Unemployment rate, opioids misuse and other substance abuse: quasi-experimental evidence from treatment admissions data. *BMC Psychiatry*. 2021/01/10 2021;21(1):22. doi:10.1186/s12888-020-02981-7
7. U.S. Census Bureau. Table DPO3 | Selected Economic Characteristics. https://data.census.gov/table/ACSDP5Y2023.DP03
8. Udalova V, Bhatia V, Polyakova M. Association of Family Income With Morbidity and Mortality Among US Lower-Income Children and Adolescents. *JAMA*. 2022;328(24):2422-2430. doi:10.1001/jama.2022.22778

© 2025 Blumenthal KG et al. *JAMA Network Open*.
