# Supplementary material for: Geospatial Socioeconomic Indicators and Penicillin Allergy Delabeling in Primary Care Patients
Source: JAMA Netw Open. 2025 Aug 22;8(8):e2528714. doi: 10.1001/jamanetworkopen.2025.28714 (PMC12374214; doi:10.1001/jamanetworkopen.2025.28714)
Supplement: Supplement 2. — Data Sharing Statement [file jamanetwopen-e2528714-s002.pdf]

Data Sharing Statement

Blumenthal. Geospatial Socioeconomic Indicators and Penicillin Allergy Delabeling in Primary Care Patients. *JAMA Netw Open*. Published August 25, 2025.
doi:10.1001/jamanetworkopen.2025.28714

Data
Data available: Yes
Data types: Deidentified participant data
How to access data: Email corresponding author Dr Kimberly G Blumenthal (kblumenthal@mgh.harvard.edu)
When available: With publication

Supporting Documents
Document types: None

Additional Information
Who can access the data: Researchers whose proposed use of the data has been approved
Types of analyses: For specified research on antibiotic allergy
Mechanisms of data availability: After approval of a proposal with a signed data access agreement
